# History of the management of talar fractures: from the fall of king Darius to Garibaldi’s bullet and from the earliest to current operative strategies

**DOI:** 10.1007/s00264-023-05766-1

**Published:** 2023-03-16

**Authors:** Carlo Biz, Alberto Crimì, Mariapaola Refolo, Felicia Deborah Zinnarello, Davide Scapinello, Mariachiara Cerchiaro, Pietro Ruggieri

**Affiliations:** Orthopedics and Orthopedic Oncology, Department of Surgery, Oncology and Gastroenterology DiSCOG, University of Padova, via Giustiniani 2, 35128 Padua, Italy

**Keywords:** Talus, Talar fractures, Trauma, Ankle, Necrosis, Osteosynthesis, Plating

## Abstract

**Purpose:**

This historical review aims to highlight the important roles of the talus in antiquity and to summarise the multiple attempts of managing talar fractures throughout history.

**Method:**

Archaeological, religious, artistic, literary, historical and scientific accounts were searched for the descriptions of talus fractures in different eras and their treatments to provide a thorough analysis of the evolution of trauma care up to the present.

**Results:**

This review shows how the talus has always had an important role in several societies: it was used as a die or considered to have a divinatory function in Mesopotamian civilisations, among Greeks and Romans, in Mongolia and in pre-Columbian Americas. Famous talus fractures are recorded in Herodotus’ Histories and in the Acts of the Apostles. We report the earliest injuries described and the first operative managements between 1600 and 1800, including the one that saved Garibaldi’s life in 1862, until the modern osteosynthesis by the first screws and nails and the current fixation by plating.

**Conclusion:**

The blooming of orthopaedic surgery at the end of nineteenth century and the high volume of traumas managed in the World Wars brought a better understanding of fracture patterns and their operative treatment. By the work of Hawkins and his classification, the introduction of the CT scan, a better knowledge of injury modalities and bone vascularisation, these challenging injuries finally land in the contemporary era without mysteries. The subsequently developed surgical procedures, although not guaranteeing success, greatly reduce the risk of necrosis and complication rate, improving patient outcomes.

## Introduction and the origin of the name

**Fig. 1:**
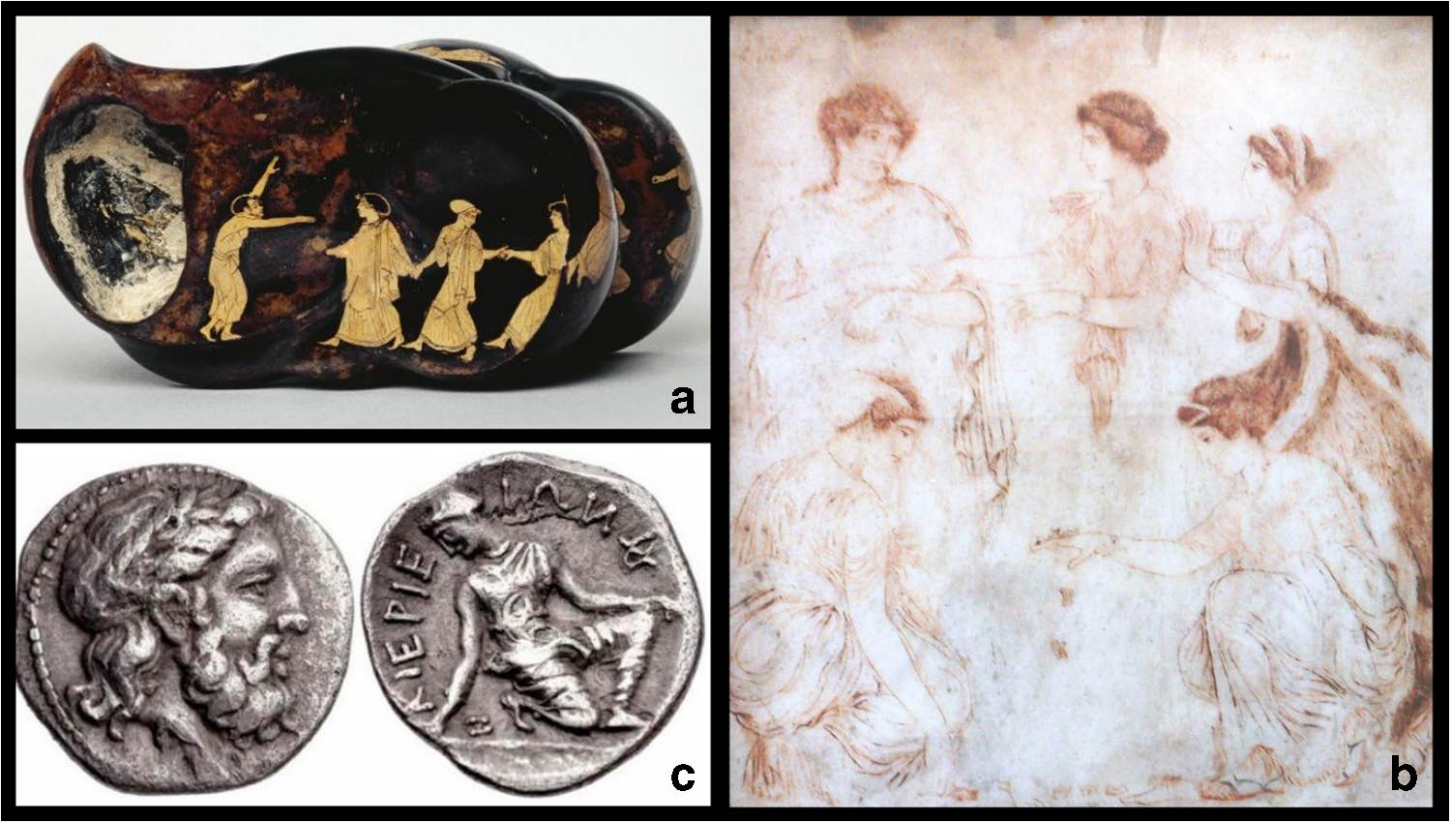
Astragals in ancient civilisations: **a** Painted attic astragalus, fifth century BC, British Museum, London; **b** players of astragals: Niobe, her daughters and Leto. Author: Alexandros of Athens. First century AD. Naples, National Archaeological Museum; **c** Diobol of Kierion, Thessaly (400–350 BC) with Zeus laureate and the Nymph Arne, kneeling and playing with astragals

The name ‘talus’ in Latin has the meaning of ‘anklebone’ and ‘die’ at the same time because the ‘knucklebones’ or anklebones, with their tetrahedral shape, were used as dice for games by Greeks and Romans [1]. In Latin literature, the word talus is first documented in the Miles Gloriosus by Plautus, where it is used in the double meaning of part of the ankle and gaming dice [2]. Also, the Mongolians and other cultures used animals’ talus bones as dice for fortune-telling [1]. Talus is also known as astragalus from the Greek ἀστράγαλος (astragalos), which means ‘dice’ but also ‘vertebra’; the second cervical vertebrae of sheep were used as dice by Greeks, after having removed the posterior arches [1] In the plural form ‘astragaloi’, it was used for the first time in the Iliad [2]. In architecture, astragalus or astragal is the part of the column in between the capital and the shaft of the column, which can be carved and is characteristic of different types of columns [3] (Fig. 1a).

In the human body, the talus is the most proximal bone of the hindfoot and lies under the tibia. It is the connection between the ankle joint and the foot, with 60% of its surface being covered by cartilage. There are no muscular origins or insertions into the talus. It rests down broadly on the calcaneus and anteriorly fits its broad head within the concavity of the scaphoid. The function of the talus is to distribute the load transmitted to it from the tibia onto the calcaneus and the scaphoid. It shows conspicuous primitive morphological changes in congenital clubfoot. In traumatology, fracture of the talus is the second most frequent fracture of the tarsal bones, the most common being heel fractures.

Talar fractures are approximately 1% of all fractures and account for 3–6% of fractures in the foot [4]. It has unique anatomy and function with multiple articulations, a large cartilaginous surface area and fragile blood supply. Short- and long-term consequences of talar fractures cause a high burden of complications and long-term disability [4]. It may be fractured by direct causes, especially by the action of gunshot bullets; but more frequently by indirect causes, as in a fall from height [5–8]. Unique types of talus fractures are the aviator fracture, described in World War I [9] and caused by a forced dorsiflexion of the foot in the impact with the rudder pedal, and the most recent snowboarder fracture, a lateral process talus fracture that occurs when a snowboarder lands after a jump with dorsiflexion of the ankle and inversion of the hindfoot [10, 11].

To the best of our knowledge, in the medical literature, there is a lack of a single report that summarises the various talus injuries and relative treatments in previous eras to the present. Hence, this narrative review aims to gather not only the main aspects concerning the relevant roles assumed by talus in antiquity, but also the famous traumatic events in which the astragalus was involved and treated over time, from the earliest managements to the most current operative strategies.

## The game of the astragals

As described before, the talus was used for centuries to play dice. The first evidence of the game of the astragals dates back to the 800 BC with some terracotta statues preserved in the British Museum. The origin of the game seems to be from Mesopotamia and, according to Herodotus [12], it was a game invented by the Lydians. It was played in Persia, Turkey and, with some variations, was still common in the 1900s, as Lovett testifies in his work of 1901 [13].

Literary sources testify the use of astragals for gaming in Greece as early as Homeric times [14]. Plutarch and Cicero [15] described the game to be common among children and women but also enjoyed by the elderly [16]. Plutarch recounts that Alcibiades played with astragals when he was a child.

The Romans used to play astragals mainly during Saturnalia, the ancient Roman festival of Saturn, held in December, a period of merrymaking and the precursor of our Christmas, where slaves sometimes even played with their masters. The game was called ‘*ludere talis*’, or playing knucklebones. Each side of the talus had a numerical value, like a die, but it was determined by the shape of the side facing up when rolled, rather than by dots indicating the value. Usually, four astragals were rolled at a time and the combination of the four faces of the four astragals made different points and the winning score.

Talus bones were also used for games such as Odd and Even, which consisted of choosing astragals randomly from a small bag or box and then guessing whether their number was odd or even.

Another game was called *Circle*, in which each player had to throw their own astragals within a circle, placed at the same distance, and move those of the opposing competitor. A further famous game, as testified by the painting by Alexander of Athens found at Resina, was the so called *Five Stones*, which consisted of throwing astragals in the air and catching them on the back of the right hand; those that fell to the ground had to be picked up during the next throw of those caught. This game is shown on a monochrome painting on marble from Herculaneum, dated to the second century AD and housed in the National Archaeological Museum in Naples (Fig. 1b).

Many artists depicted such games in their sculptures and paintings. Pliny, in his Naturalis Historia, mentions a statue by Polycletus depicting two naked boys playing knucklebones from which the statue received the name of ‘Astragalizontes’ [17].

The game of astragals is often depicted in decorated pottery. The players are children and adults, women and gods. The talus also had other functions: astragals were worn as good-luck jewellery, they were used in divination rites (throwing by the priest in front of the simulacrum of the god was one of the methods of predicting the future) and finally for weight and monetary purposes (Fig. 1c).

## First described talar fracture: the injury of Darius I

The first known talar fracture is described in the third book of Herodotus ‘Histories’; the Greek historian narrates the tale of the famous physician from Croton, Democedes, and the king of Persia, Darius I (VI sec. B.C.). During hunting, king Darius got off his horse in a rough way and seriously sprained his ankle with a dislocation of the astragalus.

The wise and famous Egyptian physicians at his court were not able to heal the shooting pain which kept him awake for seven days and seven nights, so Democedes, a Greek doctor, who was a slave at the court of Darius proposed a Greek care for the king, and this turned out to be providential.

Herodotus’ statements made the severity of the injuries reported by the Persian king clear. Specifically, the words used in the text imply a torsion movement and traumatic dislocation of the astragalus from the joint. The therapeutical approach adopted by the Egyptian physicians turned out to be ineffective; the torsion manoeuvres of the feet seemed to aggravate the clinical status, increasing the pain and depriving king Darius of his sleep, resulting in a death sentence for them. The medical approach chosen by Democedes after the mechanical, severe chiropractic treatments of the Egyptian physician was different and based on mild cures: ‘*using Greek medications and bland remedies after those tough treatments […]*’.

In general, Hippocratic texts expected for articular injuries a mechanical approach first, with extension, reduction and tight bandaging, then in succession a mild treatment with medications, diet and soft bandaging, exactly as was performed on Darius I.

Democedes asked the Persian king grace for the Egyptian colleagues who had not managed to heal him. This attitude might even conceal the awareness about the importance of the physical manoeuvres operated by the Egyptian physicians in the first phase of the treatment, although he had no hesitation taking credits for the recovery of the king. Moreover, the medical historian M. Grmek assumes that the severe pain caused by the chiropractic manoeuver was due to a fracture, which usually comes with this kind of fast, violent sprain. Those treatments, harsh and mild, which seem to be reflected in the Hippocrates texts, restored the king’s sleep, most likely by reducing the inflammation [18–22].

## Simon the Magician and the clash with the apostle Peter

**Fig. 2:**
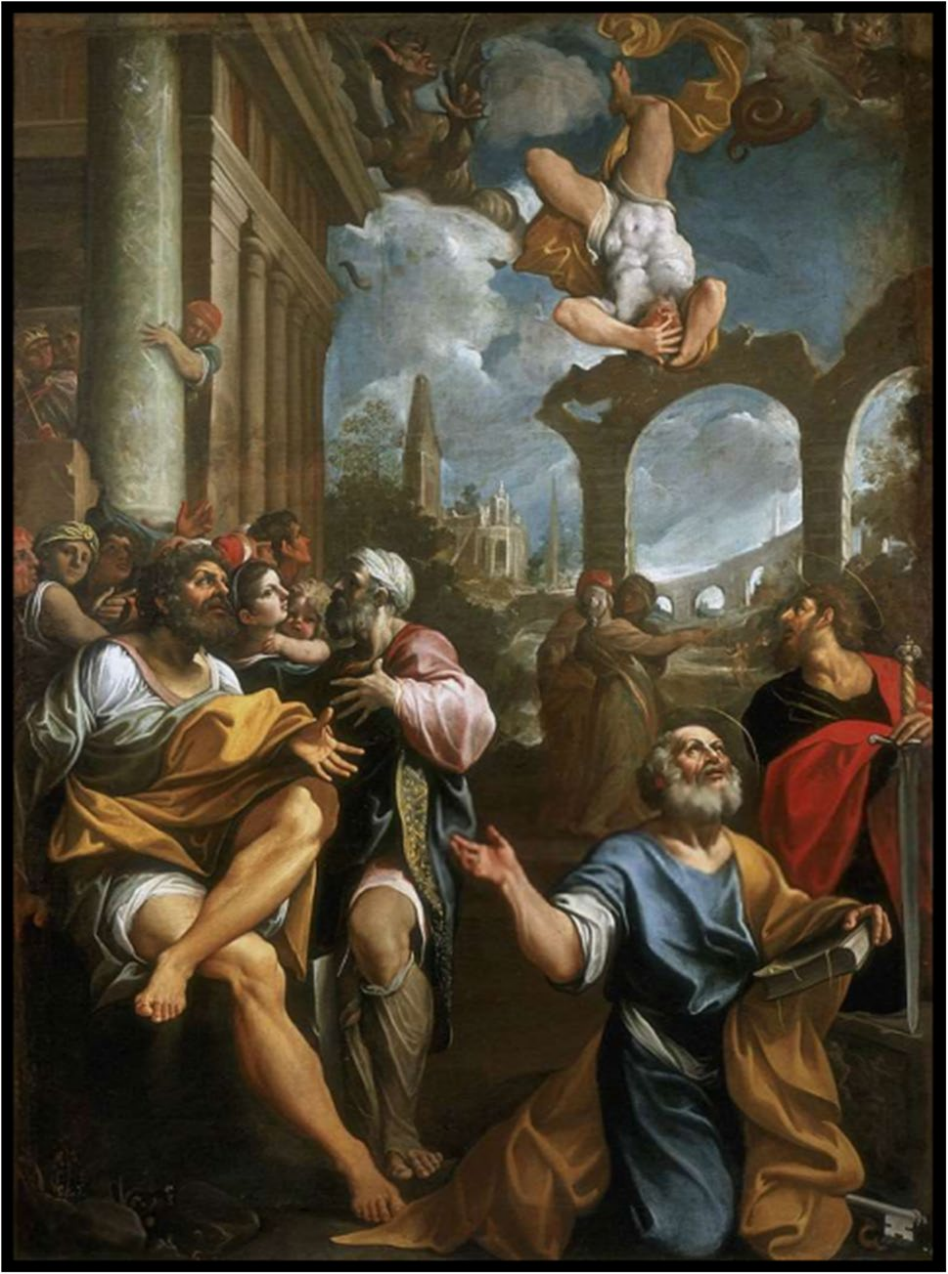
Painting by Ludovico Carracci: Caduta di Simon Mago—The Fall of Simon the Magician, 1603, oil on canvas, 342 × 265 cm, Capodimonte Museum, Italy

An episode of talar fracture is described in the Didascalia Apostolorum and affected Simon Magus, whose discourse with the apostle Peter is recorded also in Acts of the Apostles (8:9–24). The magician was considered by the early Catholic Church as the crippled anti-Christ [23]; he was the leader of a gnostic heresy in the region of Samaria in the first century after Christ [23] (Fig. 2).

According to Acts, Simon Magus, or the Magician, was a religious figure of the first century AD, who used to dazzle people of Samaria with trickery and sorceries and was then converted to Christianity by the Evangelist Philip [24].

As he was astonished by the prodigies done by the two disciples of Jesus sent to Samaria, Peter and John, especially for the power of imparting the Holy Spirit to anyone on whom they would place their hands, he offered a payment to Peter in exchange to learn this power, incurring Peter’s wrath. ‘*“Give me also this ability so that everyone on whom I lay my hands may receive the Holy Spirit.” But Peter answered: “May your money perish with you, because you thought you could buy the gift of God with money!”*’ [24]. This episode gave rise to the term ‘simony’, to describe the act of selling church offices and roles or sacred things [25].

Many other dialectical and public clashes between Peter and Simon Magus are reported in the apocryphal Acts of Peter (4–32), mostly occurring in Rome, where the Samaritan sorcerer had acquired many followers with his wonders and magic.

In Didascalia Apostolorum, another apocryphal text of the New Testament describes their final and most famous clash [26], which inspired various painters over the centuries from Cimabue (1277–1283) to Carracci (1603) and Batoni (1765). In this episode, Simon was performing magic in the Forum, and to prove himself to be a god, levitated up into the air. The apostle Peter prayed to God to stop his flying, Simon stopped mid-air and fell, breaking his ‘*anklebone*’ as reported in the English translation by R. Hugh Connolly [27], or more precisely, his ‘*astragalo*’ (talus) in the Italian version by Valentina Ragucci [28]. At that point, defeated by Peter and as reported in the Acts of Peter, the seriously injured magician was carried from Rome to Ariccia and finally to Terracina where he died ‘while being sorely cut by two physicians’ [29].

## The bullet that could have changed Italian history

There is a clear description of the difficulties and doubts in the treatment of a talar fracture in the injury occurred to the Italian hero of the Two Worlds, Giuseppe Garibaldi, in the battle of the Aspromonte the 29th of August 1862 [30]. Among the first doctors to assist the General there was Pietro Ripari, a Red Shirt who followed Garibaldi in all of his Italian expeditions from the Republic of Rome in 1849 to the attempt to conquer Rome in 1867. He wrote an interesting account about the wound of Garibaldi [31]. After being imprisoned in Varignano, Garibaldi was visited by illustrious doctors of his time, among them the Italians Francesco Rizzoli and Ferdinando Zannetti, the famous French surgeon Auguste Nèlaton, the British surgeon Richard Partridge and the Russian surgeon Nicolai Pirogoff. There was a debate about whether the bullet was still inside the wound or not. The young surgeon Enrico Albanese, who was present during the fighting and attempted a surgical incision on the field, was the first to understand that the bullet was still inside the wound, but due to his young age he was not trusted until other more famous surgeons accepted the idea of the bullet still in place. The examinations showed that the tibial malleolus was fractured at the base, but not with the comminution expected to be caused by a gun bullet going through it and into the talus [31].

**Fig. 3:**
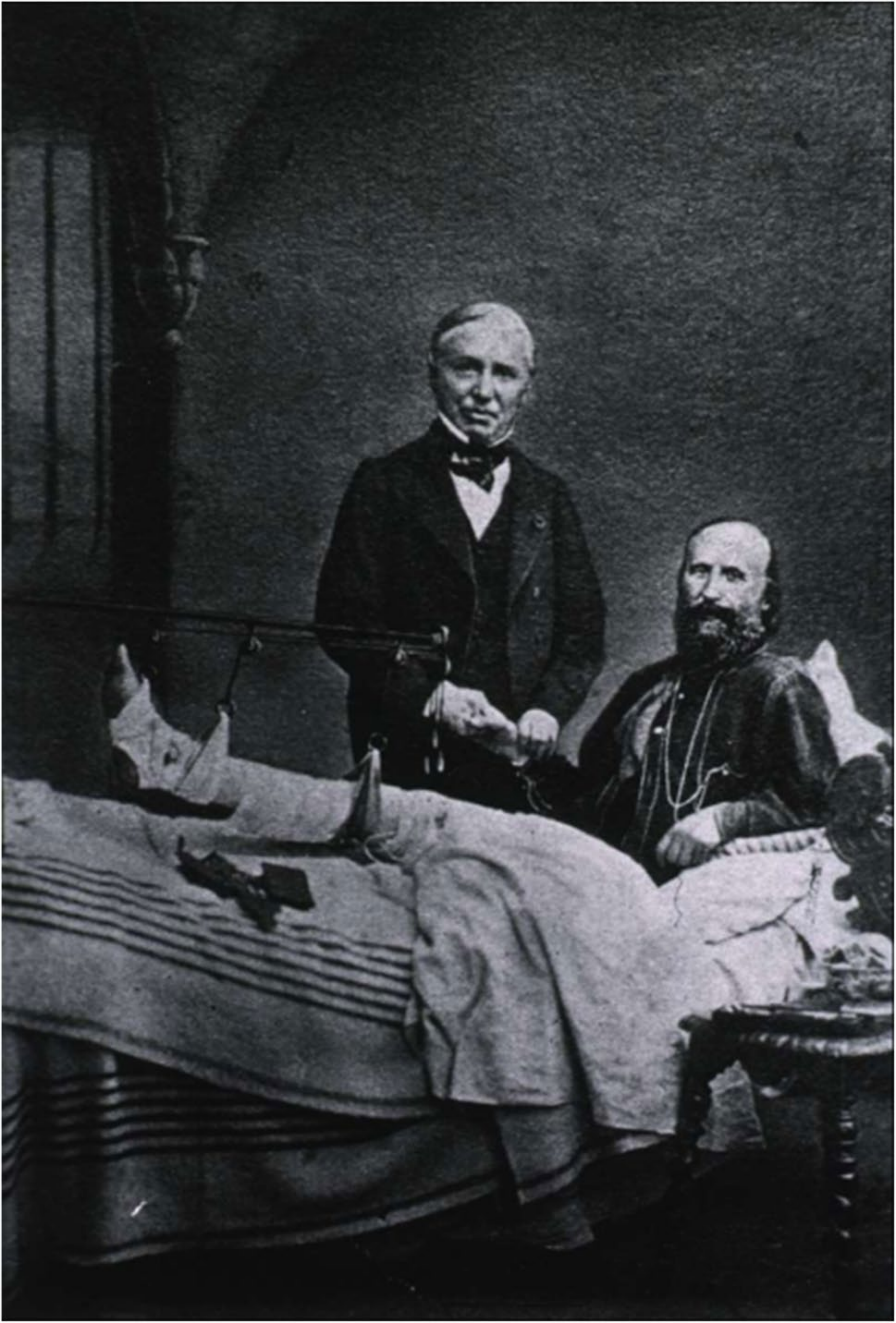
Nélaton visits Garibaldi declaring that an amputation is not necessary, hospital scene, photographer Pierre Warner

The treatment decided was so to clean the wound twice every day, with removal of fragments of bone and clothes. Partridge visited Garibaldi multiple times and brought from England a suspension device for the lower leg. An immobilisation device, Riboli’s ‘doccia’, was used that was made with small adaptable pillows. The general condition of the patient was deteriorating, infection of the wound worsened and signs of sepsis appeared [30]. A Viennese surgeon suggested a below-the-knee amputation as the only possible treatment to save Garibaldi. Hereczeghy, a Hungarian doctor friend of Garibaldi, asked help from his former teacher, the French surgeon Nèlaton, who came from Paris reassuring that, as already stated by the Italian surgeons, the limb could be saved. Examining the wound, he estimated that the conic-cylindric bullet was in the body of the talus, shown also by the great quantity of talar bone fragments in the wound. To better identify the bullet, Nèlaton suggested the use of sponges to enlarge the wound to have easier access to the fractured talus. In the following days, Garibaldi was examined by an estimated number of seventeen surgeons (Fig. 3).

The hero of the Two Worlds was said to ‘have suffered great agony’ during these exams. All of the other surgeons were still not convinced of the presence of the bullet in the fractured talus with the exception of Albanese who stated it from the very start. The scientific proof was obtained with a probe designed by Nèlaton that demonstrated the presence of lead in the fracture. It was a special probe with an unglazed porcelain tip; this absorbed and was stained by the lead in the wound confirming the presence of the bullet [32]. After almost three months and the risk of dying of sepsis, on the 23rd of November 1862, Zannetti removed the bullet from the body of the talus with forceps. The post-operative care was continued with medications and a starched bandage. Almost one year after the injury, on the 21st of August 1863, Garibaldi was declared healed from his talus open fracture [33]. He recovered the use of the lower limb, but the wound would cause intermittent pain and stiffness in the joint for the rest of his life.

## Talar fracture managements throughout history

### Historical diagnosis and treatments (1600–1900)

The first account of a talar fracture in the modern era was in the work ‘*Opera Observationum et Curationum Medico-chirurgicarum*’ of 1608 by Fabricius Hildanus [34], German barber by trade, blacksmith by birth and founding father of German surgery by acclamation. An extraordinary practitioner by today’s standards, he progressed in his job by reflecting on each case and putting into practice the principles of continuing self-education both in surgery and anatomy. His candid account of his first astragalectomy, performed in 1582, describes his patient’s recovery as very painful and long. Relief can be felt in his writing when he humbly reports the unexpected good outcome. The patient could walk.

Talar fractures, invisible as they were, almost disappeared from the medical literature until the beginning of the nineteenth century. In 1818, Cooper published a case series of talus fractures, complete with the description of each injury and clinical decision-making based on severity [35]. He recommended amputation for the very elderly, considerable tissue loss, gangrene and unacceptable deformity. Talectomy was the choice limb-salvage procedure for most fracture dislocations in the young in an attempt to avoid the necrosis and infection that usually ensued. He also described the method of closed reduction taught to him by Cline, his surgical trainer at Guy’s and St. Thomas’ Hospital in London. His work brought talar fractures to centre stage. Shortly after, in 1833, Dupuytren published his treatise ‘*On the Injuries and diseases of Bones*’, with an entire chapter on talar dislocations and their treatment [36]. Unfortunately, despite the efforts, results remained poor. A few years later, Syme reported 13 cases of talar fractures, 11 resulting in death [37]. On this basis, he recommended ankle or knee level amputation as the most effective means of avoiding morbidity. Specialist literature on the subject started flourishing, with milestones such as Monahan’s case review published in 1858 [38]. In his study, he states: ‘*the diagnosis of fracture of the astragalus is always easily made, when it is connected with an external wound communicating with the joint. But in cases of simple fracture, I cannot imagine how we can determine its existence positively, unless the fragments are displaced* […]’. The answer to his doubts appeared only in 1895 with the accidental discovery of X-ray, full of promise. The innovation did prove valuable, but not game changing, as far as talar fractures were concerned [39]. Minimally displaced fractures, compound fractures and self-reduced dislocations kept their secrets from the all-seeing X-ray eye and continued to be managed as ankle sprains [40]. Many necrosed and had to be amputated with all of the ensuing suffering, loss of mobility and impossibility to work.

Scientific tributes to talar fractures kept increasing, with names as great as Tillaux, Chaput, Destot and Labbe entering the arena. It is at this point in history that the German school gained visibility in foot and ankle surgery with many excellent studies, such as Bliesener and Gaupp’s. Around the same period, Ernst Von Bergmann ventured to perform what is thought to be the first open reduction of a fracture dislocation of the talus [41]. However, nobody followed in his steps. Irish surgeon Croly, in his 1889 paper, offers a description of closed reduction at the end of the nineteenth century: ‘*the patient should be anaesthetised, the leg flexed on the thigh, and the thigh on the pelvis, and extension made from the foot, the thumbs being applied to press back the astragalus*’ [41].

### History of modern treatments (1900–2000)

**Fig. 4:**
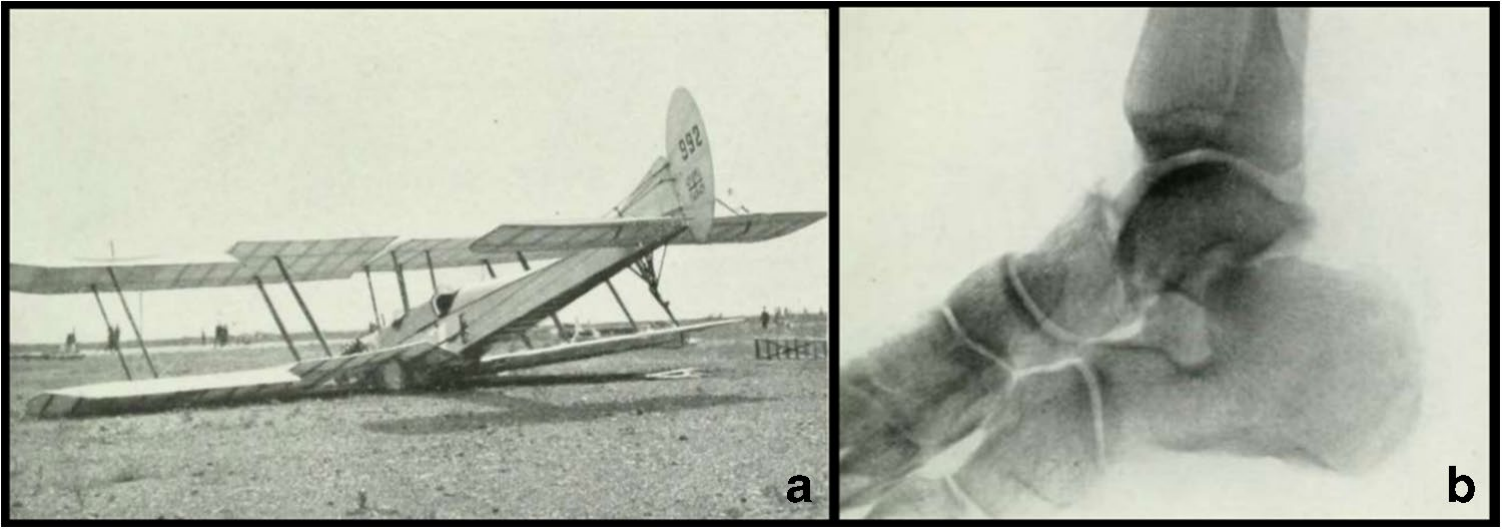
Aviator fracture: **a** example of airplane crash in the First World War; **b** pattern of talar fracture in a British aviator in the Great War

Talar excision remained the mainstay surgical treatment for complicated cases until the beginning of the twentieth century. The First World War, with the introduction of aerial warfare, provided military surgeons with new, high-energy injuries to manage. The first manual of aviation medicine appeared in 1919 by HG Anderson, consulting surgeon to the Royal Air Force. He wrote: ‘*the bone most often affected is the astragalus […] this form of injury is peculiar to aviation […] so much so that the author has ventured to name it “aviator’s astragalus*’ (Fig. 4). Diagnosis and treatment became common, and new solutions to an old problem had to be sought. Of the 18 patients in his series, 8 had been treated yet again by partial or total talectomy [42]. In the 1930s, with the publication of several important studies on fracture patho-biomechanics, the concept of accurate anatomical reduction of closed talar fractures as extolled by Miller and Baker in 1939 was the first scientifically informed attempt improving prognosis. After WWII, Anderson was succeeded by Coltart in his role as chief RAF surgical consultant. In his series of 228 talar fractures treated by Airforce surgeons, published in 1952, 106 of the most severe injuries affected combat air crash survivors. His work represents the first systematic attempt at evaluating the anatomical-descriptive classifications proposed until then, offering a pragmatic subdivision of these fractures into three families: fractures, fracture dislocations and total dislocations. It also revealed the need for a common descriptive language that could direct management. Of the historical classifications published before that, Bonnin’s [43] in 1940 and Watson Jones’ [44] in 1962 deserve mention. However, the real turning point was reached in 1970. In that year, Professor LD Hawkins of Iowa published a study on talar fractures and the risk of avascular necrosis. Based on a cohort of 57 cases, complete with X-rays, he described three main fracture patterns [45].

Hawkins’ classification remains popular to this day, having finally brought order and a sense of direction among the over-enthusiastic descriptive chaos of nineteenth century surgeons. He proved that the risk profile of talar fractures remained severe even in the face of diagnostic and technical advancement. Hawkins’ classification was further expanded by Canale, Kelly [46], Williams [47] and Vallier [48] with the addition of other subtypes of fracture patterns. To date, Hawkins’ classification remains widely employed and taught, together with the AO/ASIF/OTA classification system, that although complete, is of difficulty to use in everyday practice because of its complexity [47].

The advent of CT in the 1980s and 3D reconstruction revealed the diagnostic limits of X-rays and Hawkins’ classification. It confirmed the long-held suspicion that talar fractures are seldom isolated, and that the accurate patho-anatomy of dislocated or subluxed fractures can be extraordinarily complex [49–52]. It also elucidated the occurrence of lateral process fractures known in recent years as the ‘snowboarder’ fracture, too often underestimated by X-ray, with serious prognostic implications.

As far as treatment is concerned, Coltart reports that in the 1950s, the available options were four: conservative, open reduction, excision or fusion. In the 1970s, conservative treatment was still the mainstay for most configurations. Only the very unstable complex fracture dislocations were treated by open reduction and stabilisation with Kirschner wires in plantigrade [53, 54].

### Contemporary treatment options

Twenty years down the line, with the rise of the Scando-German school and the establishment of AO, big names such as Schatzker, Sneppen, Grob and Adelaar paved the way for the popularisation of open reduction and internal fixation (ORIF) with screws [55]. Osteosynthesis with plates and screws made its entrance in 2002 with the first review paper by a Canadian team headed by P.B. Fleuriau Chateau [56]. From then on, complex fractures have been treated surgically with a concordant drop in the number of reported complication rates and avascular necrosis [57].

Talar fractures remain a rare occurrence, yet according to recent projections, over the next 30 years, there will be a steady increase in incidence and prevalence due to the epidemic proportions of trauma, with developing countries rapidly catching up [58, 59]. Many of these lesions are still misdiagnosed as ankle sprains, leading to suboptimal treatment and significant long-term complications [60]. Timing of surgical intervention has also been re-evaluated, yet there is no definitive proof to confirm superior results with expedited surgery [61, 62].

Over the past 20 years, the traditional approach of osteosynthesis is being superseded by new generations of dedicated plates, which might offer superior results either on their own or in combination with interfragmentary compression screws. Plates have been extensively used in the management of talar neck fractures. Technological advances in plate design, particularly variable angle fixation with low profile and pliability, have opened new avenues in the management of configurations that might have required excision, such as lateral process fractures. Currently, a mini-fragment set with 2-mm and T-shaped plates, or reconstruction plates are widely available. Plates offer excellent support for different types of bone grafts. Further, mini-fragment plates offer an excellent way of treating posterior process fractures, which are often comminuted and lead to poor results. Placement of low-profile plates along the flexor hallucis longus tendon groove strikes a good balance between the need for fracture control and tendon irritation. Further studies with longer follow-ups are required to prove the effectiveness and specific long-term results and complications of new plate designs [7, 56, 63–66].

At present, there are no data to reasonably demonstrate the superiority of plates over screws only. So far, only modes of failure of either construct have been reported. Screws tend to fail in bending or by pullout, while plates break at their margins. It is thought that smaller more flexible plates might circumvent the issue in the future [63, 67–69].

## The management of talar fractures at the Orthopaedic Department of Padua University

Ever since its inception in 1957, the Orthopaedic Clinic at the University of Padua showed a consistent effort in treating and collecting results on talar fractures. Such attention was due to the increasing incidence of agriculture- and industry-related high-energy trauma in young male workers from the Venetian countryside during the post Second World War economic boom. Between 1960 and 1965, 24 cases were treated surgically. The mean patient age was 28 years. Most fractures were complex high-energy lesions with associated fractures (50%): ten cases involved subluxation of one or more fragments; concomitant fractures of the malleoli were recorded in another ten cases; body and neck fractures accounted for 60% of the sample.

**Fig. 5:**
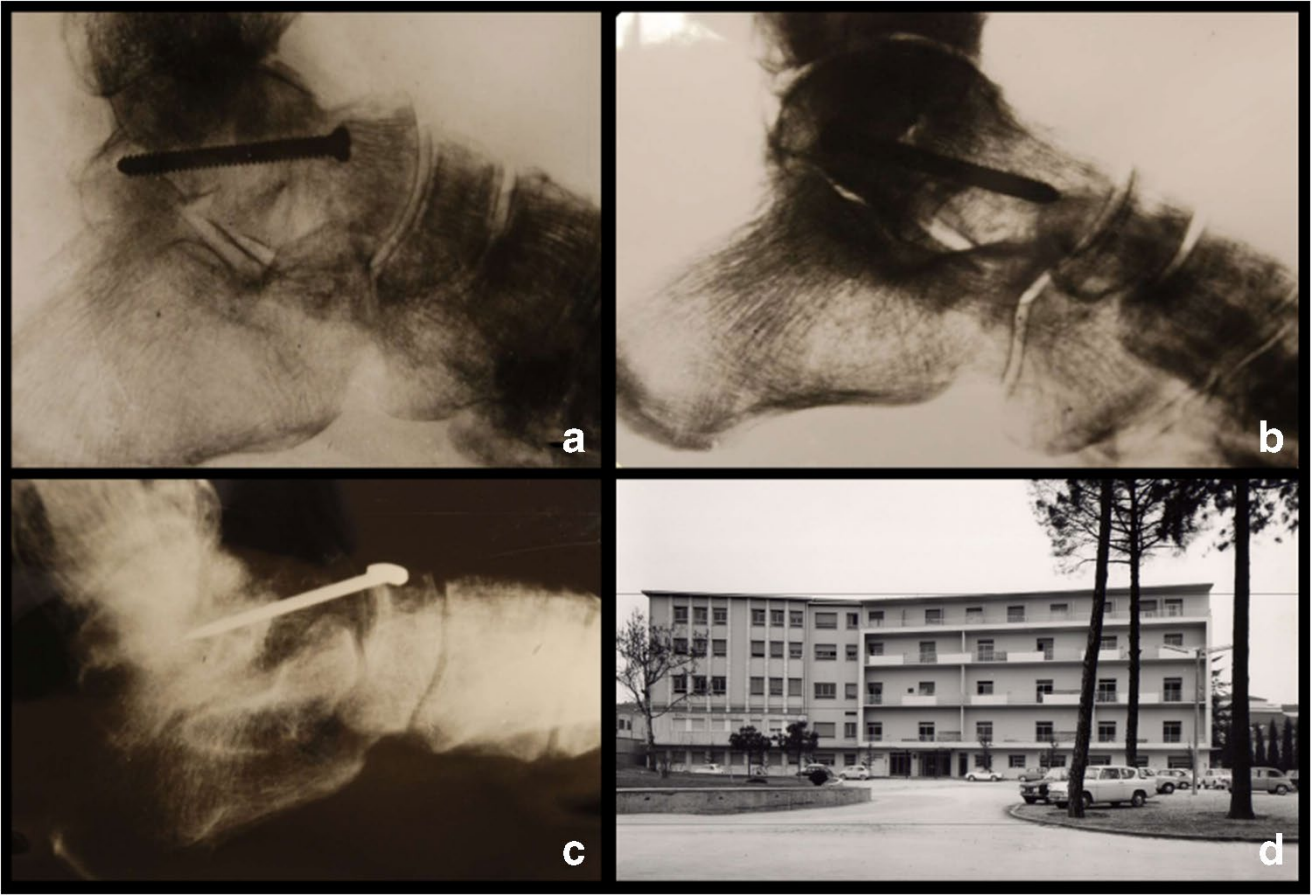
Historical pictures from the Orthopaedic Department of the University of Padova: **a**, **b** examples of internal fixation with a wood pitch screw; **c** internal fixation with a T-shaped Delitala nail; **d** Orthopaedic Department of the University of Padova in the 1960s

Regarding treatment, 13 patients underwent fixation with self-tapping pitch type screws for wood, four with Kirschner wires, one with a Delitala T screw and one with Kirschner wire and a screw. Three cases had to be revised, one by astragalectomy and two with triple arthrodesis (Fig. 5).

For that time, the results can be considered suboptimal: 15 patients reported unsatisfactory results (62.5%) and 4 had acceptable results (17%); only five satisfactory results were recorded in the sample (20.5%). A postgraduate dissertation on the topic defended in 1971 reports the following conclusion: ‘*an effective therapy is still under development due to the peculiar vascular supply which often leads to necrosis. A substitutive therapy might tackle the biological obstacle given by the insufficient regeneration capacity of such bone*’.

From the year 2000 onwards, reduction with screws had become an established trend. Of the 25 talus fractures recorded between 2003 and 2011, 20 were treated with screws, three with Kirschner wires and two with open debridement. No case of avascular necrosis was reported in the sample. The results lead to the implementation of a local protocol which included expedited surgical treatment within 24 h, anatomical reduction, fixation with at least two titanium screws, non-weight bearing for two months and an MRI scan at the three months mark to assess the risk of avascular necrosis.

The last retrospective study in 2019 by the Clinic included 31 patients treated with ORIF with at least two screws, stratified according to the Inokuchi criteria, and screened with the Hawkins’ and Sneppen classification. The progress of the cohort was measured using the visual analog scale (VAS) and the American Foot and Ankle Society (AOFAS) scores. The results showed significant rates of complications such as malunion (21.4%) and wound complications (25%), avascular necrosis (25%) and post-traumatic arthritis (78.6%). Despite this, 60% of the sample reported fair to good results. This led to confirm that, in the specific population served by the Clinic, delayed non-emergent screw fixation was a suitable option to obtain fair to good results [61].

The current institutional protocol has been modified. Emergent admission is limited to open fractures or other significant soft tissue involvement. Regardless of mechanism and energy level, all patients are offered a CT scan within 24 h. Open reduction internal fixation is delayed and organised according to soft tissue status with early involvement of an experienced plastic surgery consultant if required. Those patients that can be discharged are closely followed up in the fracture clinic every four to seven days and admitted for definitive treatment once the oedema has abated.

## Conclusions

This narrative review shows how the modern management of talus fractures is the result of more than 400 years of history (seventeenth–twenty-first century), with a global learning curve. Decision-making is informed by the general state of the patient, the number of fragments, involvement of articular surfaces, width of articular step-off, dislocations, exposure of the fracture site and soft tissue damage. Although today these injuries do not represent an orthopaedic emergency as there is not a close correlation between surgical time and risk of bone necrosis, they remain challenging to treat. Post-traumatic osteoarthritis and necrosis are the main late complications to avoid [61]. However, the surgical efforts made over the centuries still do not guarantee, either to patients or to orthopaedic surgeons, that they can be avoided with scientific certainty.

## Data Availability

Any research materials of this study are available at our institution and can be accessed.
